# Goatpox virus Hrf-063 interacts with host eIF4A1 and is associated with altered expression of antiviral signaling-related factors

**DOI:** 10.3389/fcimb.2026.1840976

**Published:** 2026-06-01

**Authors:** Baoqin Long, Huixiang Wang, Linjin Yu, Bo Liu, Mengjiao Xu, Haerleha Amantai, Weiqian Tian, Haoran Chen, Haolai Qin, Yahui Han, Longxing Shi, Jingxin Gu, Mingxuan Tang, Zhiyi Ge, Jia Chen, Youwen Li

**Affiliations:** 1 College of Animal Science and Technology, Tarim University, Tarim Animal Disease Diagnosis and Prevention Engineering Laboratory, Xinjiang Production and Construction Corps, Alaer, Xinjiang, China; 2 Key Laboratory of Livestock and Forage Resources Utilization around Tarim, Ministry of Agriculture and Rural Areas, Alaer, Xinjiang, China; 3 Provincial-Ministerial Jointly Established National Key Laboratory Cultivation Base for the Conservation and Utilization of Biological Resources in the Tarim Basin, Alaer, Xinjiang, China

**Keywords:** antiviral signaling, Capripoxvirus, eIF4A1, goatpox virus, host-virus interaction, Hrf-063, viral replication

## Abstract

Goatpox virus (GTPV) is an important capripoxvirus that causes severe disease in goats, yet the functions of many GTPV-encoded proteins remain unclear. In this study, eukaryotic translation initiation factor 4A1 (eIF4A1) was identified as a host protein interacting with GTPV Hrf-063 by GST pull-down combined with LC-MS/MS analysis. This interaction was further confirmed by reciprocal pull-down assays and co-immunoprecipitation in mammalian cells. Domain-mapping analysis showed that the N-terminal region of Hrf-063 and amino acids 211–330 of eIF4A1 were critical for their interaction, and confocal microscopy demonstrated that both proteins predominantly co-localized in the cytoplasm. Functional analyses showed that Hrf-063 was negatively associated with eIF4A1 expression during GTPV infection, while eIF4A1 modulation affected the expression of the GTPV virulence-associated protein Klp2. In addition, perturbation of Hrf-063 or eIF4A1 altered the expression of STAT1 and CAV1, and Hrf-063 regulated ISG15 and STING1 transcription in a time- and infection-dependent manner. TCID_50_ assays further showed that altered Hrf-063 or eIF4A1 expression affected infectious GTPV production. Collectively, these findings identify eIF4A1 as a host interaction partner of GTPV Hrf-063 and suggest that the Hrf-063–eIF4A1 axis is involved in viral protein expression, innate immune regulation, and infectious virus production.

## 1 Introduction

Goatpox is an economically important infectious disease of small ruminants caused by goatpox virus (GTPV), a member of the genus Capripoxvirus (Hamdi et al., 2021). As a large double-stranded DNA virus, GTPV replicates in the cytoplasm and therefore relies heavily on host cellular processes to support its replication cycle (Sprygin et al., 2022). Although poxviruses encode components required for cytoplasmic gene expression, host factors remain essential for efficient protein synthesis and for the establishment of a cellular environment favorable for infection (Lu and Zhang, 2020).

Host translational regulation has emerged as an important interface in virus-host interactions. Viral infection often reshapes the host translational landscape not only to promote viral protein synthesis but also to modulate the expression of immune-related molecules (Hoang et al., 2021; Rozman et al., 2023). For cytoplasmic DNA viruses in particular, the ability to engage host translation-associated factors may be closely linked to both replication efficiency and immune evasion (Chathuranga et al., 2021). Thus, identifying translation-related host targets of GTPV proteins may provide insight into how the virus coordinates replication with host antiviral responses.

Eukaryotic translation initiation factor 4A1 (eIF4A1) is a DEAD-box RNA helicase and a core component of the eIF4F complex. It plays a central role in cap-dependent translation initiation by unwinding structured mRNA regions and facilitating ribosomal scanning (Gulay et al., 2020; Taroncher-Oldenburg et al., 2021). Beyond its canonical role in translation, eIF4A1 has also been implicated in selective mRNA translation and infection-associated cellular responses, making it a plausible regulatory node during virus infection (Hernández and Vazquez-Pianzola, 2005). However, whether host eIF4A1 is directly targeted by GTPV proteins has remained unclear.

Hrf-063 is a GTPV-encoded putative host-range factor whose biological role has not yet been systematically characterized. Although poxvirus host-range genes are broadly linked to host tropism and immune modulation, the host interaction partners of Hrf-063 and its contribution to antiviral signaling regulation remain poorly defined. Given the dependence of poxvirus replication on host translational machinery and the regulatory relevance of eIF4A1 (Lu and Zhang, 2020; Rozman et al., 2023; Yang et al., 2024), we hypothesized that Hrf-063 may engage host translation-related machinery and thereby influence antiviral signaling-related factor expression.

In the present study, we identified eIF4A1 as a host interactor of Hrf-063 using GST pull-down coupled with mass spectrometry and validated this interaction by biochemical assays *in vitro* and in cells. We further combined structural prediction, truncation analysis, and confocal microscopy to characterize the interaction interface and subcellular localization. Finally, we examined whether Hrf-063 and eIF4A1 were associated with altered expression of antiviral signaling-related factors, including STAT1, CAV1, *ISG15*, and *STING1*. Our findings provide evidence that Hrf-063 targets eIF4A1 and suggest a potential link between this interaction and the remodeling of host antiviral responses during GTPV infection.

## 2 Materials and methods

### 2.1 Cells, virus, plasmids, and antibodies

Vero and OA3.Ts cells were maintained at the Key Laboratory of Tarim Animal Husbandry Science and Technology Corps and cultured in Dulbecco’s Modified Eagle Medium (DMEM; KeyGEN, Jiangsu, China) supplemented with 10% fetal bovine serum (FBS), 100 U/mL penicillin, 100 μg/mL streptomycin, and 0.25 μg/mL amphotericin B at 37 °C in a humidified incubator with 5% CO_2_.

GTPV strain AV41 was propagated and titrated in goat-derived cells and stored at -80 °C until use. Viral titers were determined by plaque assay, and infections were performed at a multiplicity of infection (MOI) of 1.

Primary antibodies against GST, His, Flag, and HA tags, as well as all secondary antibodies, were purchased from TransGen Biotech (Beijing, China). Primary antibodies against GAPDH, STAT1, and CAV1 were obtained from Proteintech (Wuhan, China), while antibodies against Hrf-063 and eIF4A1 were sourced from Biodragon (Suzhou, China). GST agarose beads and protein A/G agarose beads were purchased from TransGen Biotech (Beijing, China) and Santa Cruz Biotechnology (Santa Cruz, CA, USA), respectively. All other reagents were of analytical grade unless otherwise specified.

### 2.2 Construction of recombinant plasmids and truncated mutants

The coding sequences of *Hrf-063* and host *eIF4A1* were amplified by PCR using gene-specific primers and subsequently cloned into appropriate prokaryotic or eukaryotic expression vectors. For bacterial expression, the full-length coding sequences of *Hrf-063* and *eIF4A1* were inserted into pGEX-KG and pET-42b(+) vectors to generate the recombinant plasmids pGEX-Hrf-063 and pET-His-eIF4A1, respectively.

For co-immunoprecipitation (Co-IP) assays, the full-length coding sequences were cloned into mammalian expression vectors containing Flag or HA epitope tags, resulting in the constructs pcDNA3.1-Flag-Hrf-063 and pcDNA3.1-HA-eIF4A1. For subcellular localization analysis, Hrf-063 and eIF4A1 were fused to EGFP and DsRed tags, respectively.

To identify the interaction domains, multiple truncated mutants of Hrf-063 and eIF4A1 were designed and constructed based on their sequence characteristics and structural predictions. PCR-amplified fragments corresponding to the indicated regions were cloned into the same expression vectors as their full-length counterparts. All recombinant constructs were verified by restriction enzyme digestion and Sanger sequencing prior to use (**Table 1**).

**Table 1:** Primer sequences used in the construction of recombinant plasmids.

Gene name	Primer sequence (5′ to 3′)
pGEX-Hrf063	F: CGGGATCCATGGGTATCAGACACGAG R: CCCAAGCTTTTATTCATAGTCATCCTCTAT
pET-His-eIF4A1	F: ATGTCTGCGAGTCAGGAT R: CCGCTCGAG GATGAGGTCAGCAACATTG
pcDNA3.1-eIF4A1	F: CCCAAGCTTGCCACCTACCCATACGATGTTCCAGATTACGCTATGTCTGCGAGTCAGGATTCCC R: CCTCGAGGGATGAGGTCAGCAACATTGAGGGG
pcDNA3.1-Hrf-063	F: CCCAAGCTTGCCACCGATTACAAGGATGACGACGATAAGATGGGTATCAGACACGAGTTAGATATTTTG R: CCTCGAGGTTCATAGTCATCCTCTATATCATTAAC
GST-Hrf-063-1	F: CGCGGATCCATGGGTATCAGACACGAGTTAGATATTTTGC R: CCAAGCTTggTAATATAATAATAAAATCCAATTTTTTTTGTTGA
GST-Hrf-063-2	F: CGGGATCCATGAAGCGTTATTACGAAATAAAAGATTCAAGAATGAC R: CCAAGCTTggTCATTCATAGTCATCCTCTATATCATTAACTTCG
GST-Hrf-063-3	F: CGGGATCCATGAAAGATTACATAAATATAAGTGATGATTATTATCTGTATGATGC R: CCAAGCTTggTTCATAGTCATCCTCTATATCATTAACTTCGTCG
His-eIF4A1-1	F: CGCCATATGATGCTGGCACCCACTAGAGAGTTGG R: CCGCTCGAGGATGAGGTCAGCAACATTGAGGG
His-eIF4A1-2	F::CGCCATATGATGTTGCTGTCAGCTACAATGCCTTC R: CCGCTCGAGGATGAGGTCAGCAACATTGAGGG
His-eIF4A1-3	F: CGCCATATGATGGACCTACTGGCCAGAGGCA R: CCGCTCGAGGATGAGGTCAGCAACATTGAGGG
pEGFP-Hrf-063	F: CCCAAGCTTGCCACCATGGGTATCAGACACGAGTTAGATATTTTG R: CGGGATCCCGTTCATAGTCATCCTCTATATCATTAACTTC
DsRed-eIF4A1	F: CCGCTCGAGGCCACCATGTCTGCGAGTCAGGATTCC R: CGGGATCCCGGATGAGGTCAGCAACATTGAGGGG

### 2.3 GST pull-down screening and mass spectrometry

To identify host proteins that interact with Hrf-063, the recombinant plasmid pGEX-Hrf-063 was transformed into Escherichia coli BL21 cells, with the empty pGEX vector transformed in parallel as the GST control. Protein expression was induced with 1 mM isopropyl β-D-1-thiogalactopyranoside (IPTG) at 37 °C for 6 h. Bacterial cells were harvested and lysed, and the clarified lysates were subjected to affinity purification.

GST-Hrf-063 and GST control proteins were purified using ProteinIso GST Resin (TransGen Biotech, China) according to the manufacturer’s instructions. Briefly, the lysates were loaded onto resin columns pre-equilibrated with phosphate-buffered saline (PBS, pH 7.3), followed by extensive washing to remove unbound proteins. The bound proteins were eluted with 50 mM Tris-HCl buffer (pH 8.0) containing 10 mM reduced glutathione. The expression and purity of the GST-tagged proteins were evaluated by sodium dodecyl sulfate-polyacrylamide gel electrophoresis (SDS-PAGE).

For GST pull-down assays, purified GST-Hrf-063 and GST control proteins were immobilized on glutathione agarose beads and incubated with whole-cell lysates prepared from OA3.Ts cells at 4°C for 12 h. After incubation, the beads were washed extensively to remove non-specifically bound proteins. The bound proteins were then eluted and separated by SDS-PAGE.

Differential protein bands were subjected to liquid chromatography-tandem mass spectrometry (LC-MS/MS) for protein identification. Candidate Hrf-063-interacting proteins were identified based on peptide-matching results. Among the identified candidates, eIF4A1 was selected for further validation because of its relatively high peptide coverage and mass spectrometry score.

### 2.4 *In vitro* pull-down and co-immunoprecipitation assays

For *in vitro* pull-down validation, GST-Hrf-063 and GST control proteins expressed from pGEX-Hrf-063 and pGEX-KG, respectively, were immobilized on glutathione agarose beads and incubated with purified His-eIF4A1 at 4 °C for 12 h. After extensive washing, the bound proteins were eluted and analyzed by sodium dodecyl sulfate-polyacrylamide gel electrophoresis (SDS-PAGE) and Western blotting using anti-His or other indicated antibodies.

For co-immunoprecipitation (Co-IP) assays, cells were co-transfected with plasmids expressing Flag-Hrf-063 and HA-eIF4A1 using Lipofectamine™ 2000 reagent (Invitrogen; Thermo Fisher Scientific, Inc., Cat. No. 11668019) according to the manufacturer’s instructions. At 36 h post-transfection, cells were lysed in immunoprecipitation lysis buffer supplemented with protease inhibitors. The clarified lysates were incubated with anti-Flag or anti-HA antibodies together with protein A/G agarose beads at 4 °C overnight. After washing, the immunoprecipitated proteins were eluted by boiling in SDS loading buffer and analyzed by Western blotting. Reciprocal Co-IP assays were performed to further confirm the intracellular association between Hrf-063 and eIF4A1.

### 2.5 AlphaFold-based structural prediction and interaction-domain mapping

To investigate the potential interaction mode between Hrf-063 and eIF4A1, structural predictions were performed using AlphaFold and AlphaFold-Multimer (Evans et al., 2021). The predicted complex model was used for preliminary analysis of the putative binding interface and to guide the design of truncation constructs.

To map the regions required for the Hrf-063-eIF4A1 interaction, full-length and truncated forms of *Hrf-063* and *eIF4A1* were generated and analyzed using GST pull-down assays. Truncated Hrf-063 constructs were examined for their ability to bind eIF4A1, whereas truncated eIF4A1 variants were evaluated for their interaction with Hrf-063. Binding was assessed by Western blotting following GST pull-down. The interaction regions were defined according to the retention or loss of detectable binding signals in the corresponding truncation mutants.

### 2.6 Immunofluorescence staining and confocal microscopy

For subcellular localization analysis, cells were seeded onto sterile glass coverslips and transfected with plasmids expressing EGFP-Hrf-063, DsRed-eIF4A1, or the corresponding empty vectors. At 24 h post-transfection, cells were washed with phosphate-buffered saline (PBS), fixed with pre-chilled 4% paraformaldehyde for 25 min, and washed again with PBS. Cell nuclei were counterstained with DAPI.

Fluorescence signals were visualized using a confocal laser scanning microscope (Nikon, Japan; A1R HD25/N-SIM). Images were acquired using identical acquisition settings within each experiment. The subcellular distribution of Hrf-063 and eIF4A1 was assessed qualitatively, and colocalization was evaluated based on the overlap of fluorescence signals in merged images.

### 2.7 siRNA knockdown, overexpression, and viral infection assays

For gene knockdown experiments, cells were transfected with siRNAs targeting Hrf-063 or eIF4A1, or with the corresponding negative control siRNA, using Lipofectamine™ 2000 reagent according to the manufacturer’s instructions. For overexpression experiments, cells were transfected with the indicated recombinant plasmids expressing Hrf-063 or eIF4A1. At 24 h post-transfection, cells were either harvested directly or infected with GTPV at a MOI of 1.

For viral infection assays, cells were inoculated with GTPV for 2 h. The inoculum was then removed and replaced with fresh medium containing 2% fetal bovine serum (FBS). Cell samples were collected at 6, 12, 24, 36, and 48 h post-infection to prepare total protein extracts and total cellular RNA. Mock-infected cells were included as negative controls.

These experiments were performed to evaluate the effects of Hrf-063 and eIF4A1 knockdown or overexpression on the expression of host antiviral signaling-related factors under infected and uninfected conditions (**Table 3**).

**Table 2:** Sequences of primers used in the gene expression analysis.

Gene name	Primer sequence (5′ to 3′)
Hrf-063	F: TGCACTAAAGAATGTTGAACTTCTTAAAGG R: GTTGTATCAATAACAACACCGTTACATACC
eIF4A1	F: TGACACTGGAGGGTATCCGT R: TTCTCGGTGAGCCAATCCAC
ISG15	F: CTTGTCCACCAGGGCCTGAAAGC R: CTGCGACCCTTGTCGTTCCTCAC
CAV1	F: AGGCTATGGCAGAGGAAATGAACG R: GAAAGAGAGAATGGCAAAGTAAAT
STAT1	F: TCTGTCCTTCTTCCTGAACCCACC R: CGCTCCTTGCTAATAAAGCCCACA
GAPDH	F: AGGTCGGTGTGAACGGATTTG R: TGTAGACCATGTAGTTGAGGTCA
STING1	F: CCTGTCATCTTCCAGAAACAACCT R: CAGGAGAGCAAGCATCCAAGTGAA

**Table 3:** siRNA sequences used for gene silencing.

Gene name	Primer sequence (5′ to 3′)
si-Hrf063	F: CGACGAAGUUAAUGAUAUA R: UAUAUCAUUAACUUCGUCG
si-eIF4A1	F: AGAUUGAAUUAGAUCUAAA R: UUUAGAUCUAAUUCAAUCU
siNC	F: UUCUCCGAACGUGUCACGUTT R: ACGUGACACGUUCGGAGAATT

### 2.8 RNA extraction and RT-qPCR analysis

Total RNA was extracted from cells using the TransZol Up Plus RNA Kit according to the manufacturer’s instructions. RNA concentration and purity were determined using a spectrophotometer. Equal amounts of total RNA were reverse-transcribed into complementary DNA (cDNA) using a reverse transcription kit (Applied Biological Materials Inc., Canada; Cat. No. G592).

Quantitative real-time PCR (RT-qPCR) was performed using SYBR Green Master Mix on a QuantGene 9600 real-time PCR system (Hangzhou Borer Technology Co., Ltd., Hangzhou, China). The mRNA levels of *eIF4A1*, *STAT1*, *CAV1*, *ISG15*, *STING1*, and *Hrf-063* were measured using gene-specific primers. Relative mRNA expression levels were calculated using the 2^−ΔΔCt method (Livak and Schmittgen, 2001) with *GAPDH* used as the internal reference gene. The primer sequences are listed in **Table 2**.

### 2.9 Western blot analysis

Total cellular proteins were extracted using lysis buffer supplemented with protease inhibitors. Protein concentrations were determined using a bicinchoninic acid (BCA) protein assay kit. Equal amounts of protein were separated by sodium dodecyl sulfate-polyacrylamide gel electrophoresis (SDS-PAGE) and transferred onto polyvinylidene fluoride (PVDF) membranes.

The membranes were blocked with 5% skim milk for 4 h and then incubated with the indicated primary antibodies at 4 °C for 3 h. After washing, the membranes were incubated with horseradish peroxidase (HRP)-conjugated secondary antibodies at room temperature for 1 h. Protein bands were visualized using an enhanced chemiluminescence (ECL) detection system. The protein levels of eIF4A1, STAT1, CAV1, and the indicated tagged proteins were analyzed, with GAPDH used as the loading control. Band intensities were quantified using ImageJ software.

### 2.10 Viral harvest and TCID_50_ assay

To evaluate the effects of Hrf-063 and eIF4A1 perturbation on GTPV replication, virus-containing samples were collected from infected cells after the indicated treatments. cells were transfected with plasmids expressing Hrf-063 or eIF4A1, or with siRNAs targeting Hrf-063 or eIF4A1, followed by GTPV infection at a multiplicity of infection (MOI) of 1. At the indicated time point post-infection, both cells and culture supernatants were harvested together and subjected to three freeze–thaw cycles at −80 °C to release intracellular viral particles. The lysates were clarified by centrifugation, and the resulting virus-containing supernatants were collected for titration.

Viral titers were determined using the 50% tissue culture infectious dose (TCID_50_) assay. Virus-containing supernatants were serially diluted 10-fold from 10^−1 to 10^−9 in maintenance medium and then inoculated onto 96-well plates containing cells at approximately 90% confluence. After incubation, cytopathic effects were observed, and TCID_50_ values were calculated using the Kärber method. Viral titers were expressed as lgTCID_50_.

### 2.11 Statistical analysis

All experiments were performed with at least three independent biological replicates unless otherwise stated. Data are presented as the mean ± standard deviation (SD), as indicated in the corresponding figure legends. Statistical analyses were performed using GraphPad Prism software. Differences between two groups were analyzed using Student’s t-test, whereas comparisons among multiple groups were performed using one-way analysis of variance (ANOVA) followed by an appropriate *post hoc* test. A p value < 0.05 was considered statistically significant.

## 3 Results

### 3.1 Identification of eIF4A1 as a candidate host factor interacting with GTPV Hrf-063

**Figure 1:**
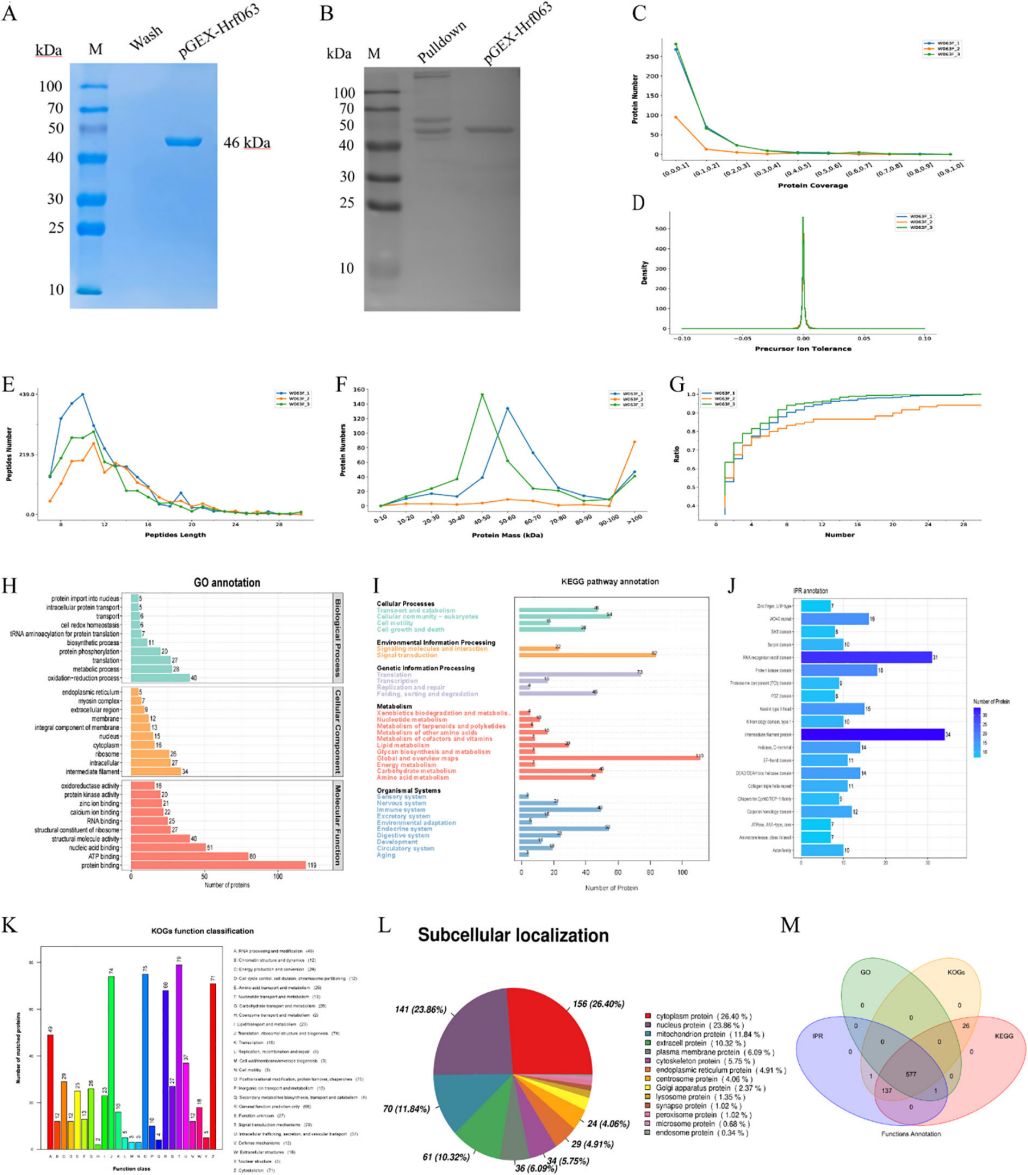
Proteomic screening identifies eIF4A1 as a host interaction partner of GTPV Hrf-063 **(A)** The Coomassie-stained SDS-PAGE gel shows that the pGEX-Hrf063 protein was successfully purified. The molecular weight marker (kDa) is shown on the left.**(B)** Coomassie-stained SDS-PAGE gel showing proteins specifically enriched in GST-Hrf-063 pull-down samples. Molecular weight markers (kDa) are indicated on the left. **(C–G)** Quality control and mass spectrometry analysis of proteins identified in GST-Hrf-063 pull-down samples. **(C)** Distribution of protein coverage **(D)** Precursor ion mass tolerance distribution. **(E)** Peptide length distribution. **(F)** Protein mass distribution. **(G)** Cumulative protein identification curve **(H)** Gene Ontology (GO) functional annotation of identified proteins, categorized into biological process, cellular component, and molecular function. **(I)** KEGG pathway enrichment analysis of interacting proteins. **(J)** Domain annotation analyses of selected candidate proteins. **(K)** KOG functional classification of interacting proteins. **(L)** Subcellular localization distribution of identified host proteins. **(M)** Overlap of functional annotations among GO, KOG, IPR, and KEGG databases.

To identify host proteins associated with GTPV Hrf-063, a GST-Hrf-063 fusion protein was constructed, expressed, and purified for GST pull-down screening. SDS-PAGE analysis showed an inducible protein band of approximately 46 kDa (**Figure 1A**), which was consistent with the predicted molecular weight of GST-Hrf-063. The purified GST-Hrf-063 fusion protein exhibited adequate purity for subsequent interaction screening.

GST pull-down assays using goat-derived cell lysates revealed three protein bands that were specifically enriched in the GST-Hrf-063 group compared with the GST control group (**Figure 1B**). These differential bands were excised and subjected to liquid chromatography-tandem mass spectrometry (LC-MS/MS) analysis for protein identification. Quality assessment of the proteomic data showed appropriate peptide length distribution (**Figure 1C**), precursor mass error distribution (**Figure 1D**), and cumulative protein identification profiles (**Figures 1F, G**), supporting the reliability of the LC-MS/MS dataset.

Functional annotation analysis showed that the identified proteins were mainly associated with translation-related biological processes, RNA binding, ATP binding, and cytoplasmic or ribosome-associated cellular localization (**Figures 1H–M**). In total, 365 proteins were identified by LC-MS/MS, and representative candidate proteins are listed in **Table 4**. Based on an integrated assessment of amino acid sequence coverage, peptide-spectrum matches, the number of unique peptides, and protein scores, eIF4A1 was ranked as the top candidate Hrf-063-interacting host protein and was therefore selected for further validation (**Table 4**).

**Table 4:** Identification of GST pull-down interacting proteins by mass spectrometry analysis.

Accessions	MW (kDa)	Coverage (%)	PSMs	Unique peptides	Score
XP_012041059.1	46.1	28	23	10	49.49
XP_027823079.1	62.4	27	28	10	46.95
XP_004007048.2	51.1	24	16	13	40.56
XP_027830327.1	50.3	23	19	2	39.94
XP_004012951.2	48.8	24	20	1	37.19
XP_004004198.2	44.9	20	11	8	32.45
XP_027832136.1	74.1	22	19	15	32.23
XP_004010269.2	44.1	21	12	9	28.92
XP_027828084.1	50.1	21	14	8	26.94
XP_004002582.1	43.0	22	13	8	25.50
XP_004006644.2	43.8	25	17	9	25.03
NP_001009467.1	52.9	20	14	9	23.98
XP_004014356.2	46.0	19	10	7	22.36
XP_004020149.1	46.4	20	14	9	21.84
XP_042095290.1	50.7	22	10	8	21.51
XP_027816604.1	42.1	24	9	8	21.31
XP_014959644.2	43.1	24	14	9	20.61
XP_027833599.1	45.7	24	11	7	20.01
XP_004006633.1	51.7	20	11	9	19.31
XP_004020196.1	42.6	23	9	3	17.53
XP_004022233.1	29.6	19	10	6	15.53
XP_011953279.1	32.7	20	7	6	12.99
XP_004008711.1	66.4	18	9	7	9.97
XP_004016949.1	48.0	23	11	8	7.45

### 3.2 Hrf-063 interacts with eIF4A1 *in vitro* and in mammalian cells

**Figure 2:**
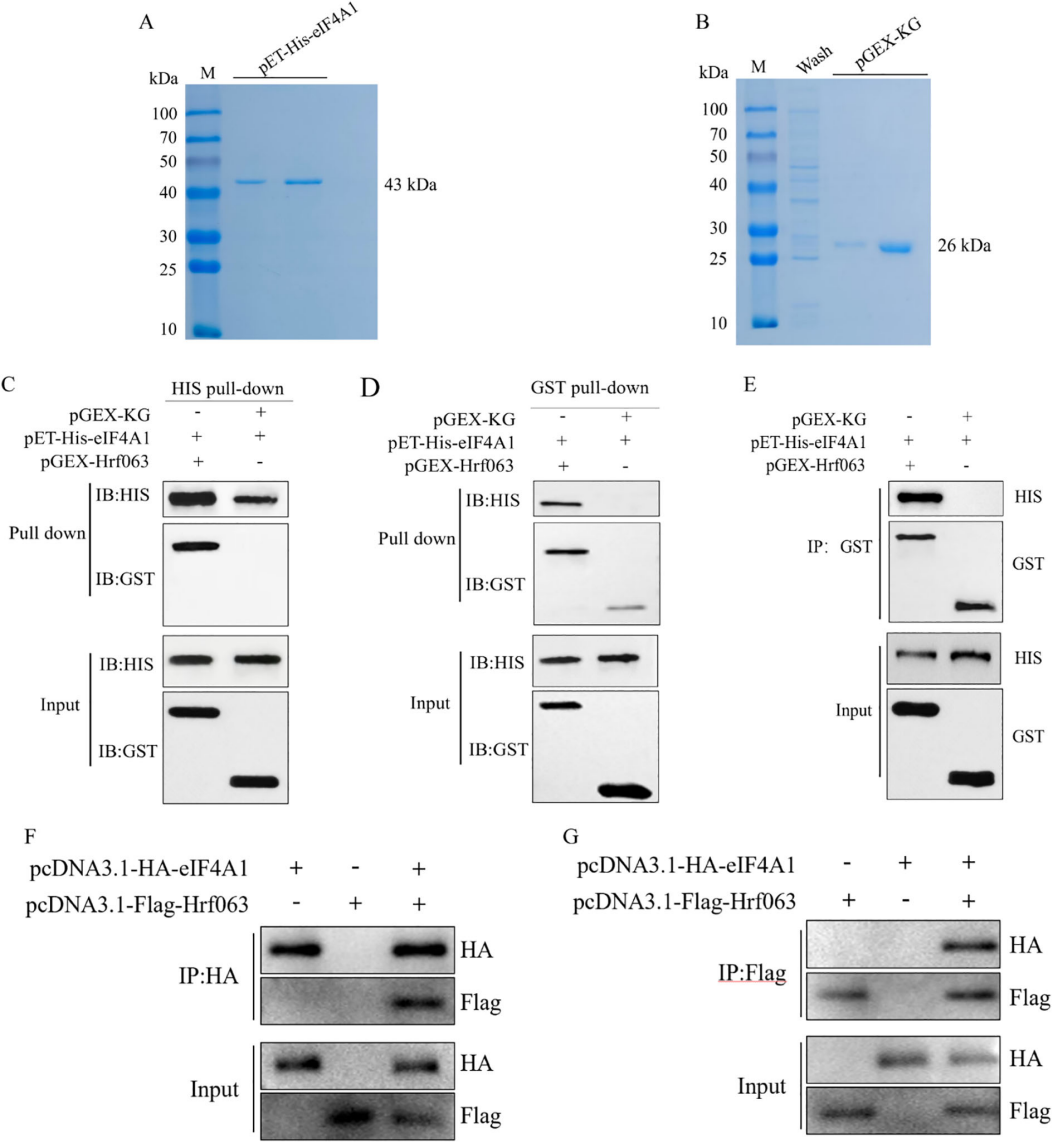
Interaction between Hrf-063 and eIF4A1 demonstrated by pull-down and co-immunoprecipitation assays. **(A)** Expression and purification of recombinant His-tagged eIF4A1 (His-eIF4A1, 46 kDa) analyzed by SDS-PAGE. M, protein molecular weight marker. **(B)** SDS-PAGE was used to analyze the expression and purification of the GST-tagged empty vector (pGEX-KG, 26 kDa). M, protein molecular weight marker. **(C)** His pull-down assay showing the interaction between GST-Hrf-063 and His-eIF4A1. **(D)** GST pull-down assay further confirming the interaction between GST-Hrf-063 and His-eIF4A1. **(E)** The co-immunoprecipitation (Co-IP) assay demonstrated an extracellular interaction between Hrf-063 and eIF4A1, which was expressed in prokaryotes. **(F)** Co-IP analysis revealed an interaction between Flag-Hrf063 and HA-eIF4A1 in Vero cells. **(G)** Reverse co-immunoprecipitation assays confirmed the interaction between Flag-eIF4A1 and Hrf-063 in Vero cells.

To validate the mass spectrometry results, recombinant His-tagged eIF4A1 and the GST-tagged empty vector pGEX-KG were expressed and purified (**Figures 2A, B**). The interaction between eIF4A1 and pGEX-Hrf063 was further examined by *in vitro* GST pull-down, reciprocal His pull-down, and co-immunoprecipitation (Co-IP) assays. pGEX-Hrf063 was detected in the pET-His-eIF4A1 pull-down group, whereas no signal was observed in the control group (**Figure 2C**). Likewise, pET-His-eIF4A1 was detected in the pGEX-Hrf063 pull-down group but not in the GST control group (**Figure 2D**). Moreover, pGEX-Hrf063 was successfully captured when pET-His-eIF4A1 was used as the bait protein, but not in the control group (**Figure 2E**). These findings demonstrated that eIF4A1 specifically interacted with Hrf063 under *in vitro* conditions.

To further investigate the interaction between Hrf-063 and eIF4A1 in mammalian cells, pcDNA3.1-Flag-Hrf-063 and pcDNA3.1-HA-eIF4A1 were co-expressed in Vero cells, followed by co-immunoprecipitation (Co-IP) analysis. When pcDNA3.1-HA-eIF4A1 was used as the bait protein, pcDNA3.1-Flag-Hrf-063 was successfully co-immunoprecipitated, whereas no corresponding signal was detected in the control groups (**Figure 2F**). Conversely, pcDNA3.1-HA-eIF4A1 was detected only when pcDNA3.1-Flag-Hrf-063 was used as the bait protein, but not in the control groups (**Figure 2G**). These results demonstrated that Hrf-063 interacted with eIF4A1 in a cellular context.

### 3.3 Identification of the interaction domain between Hrf-063 and eIF4A1

**Figure 3:**
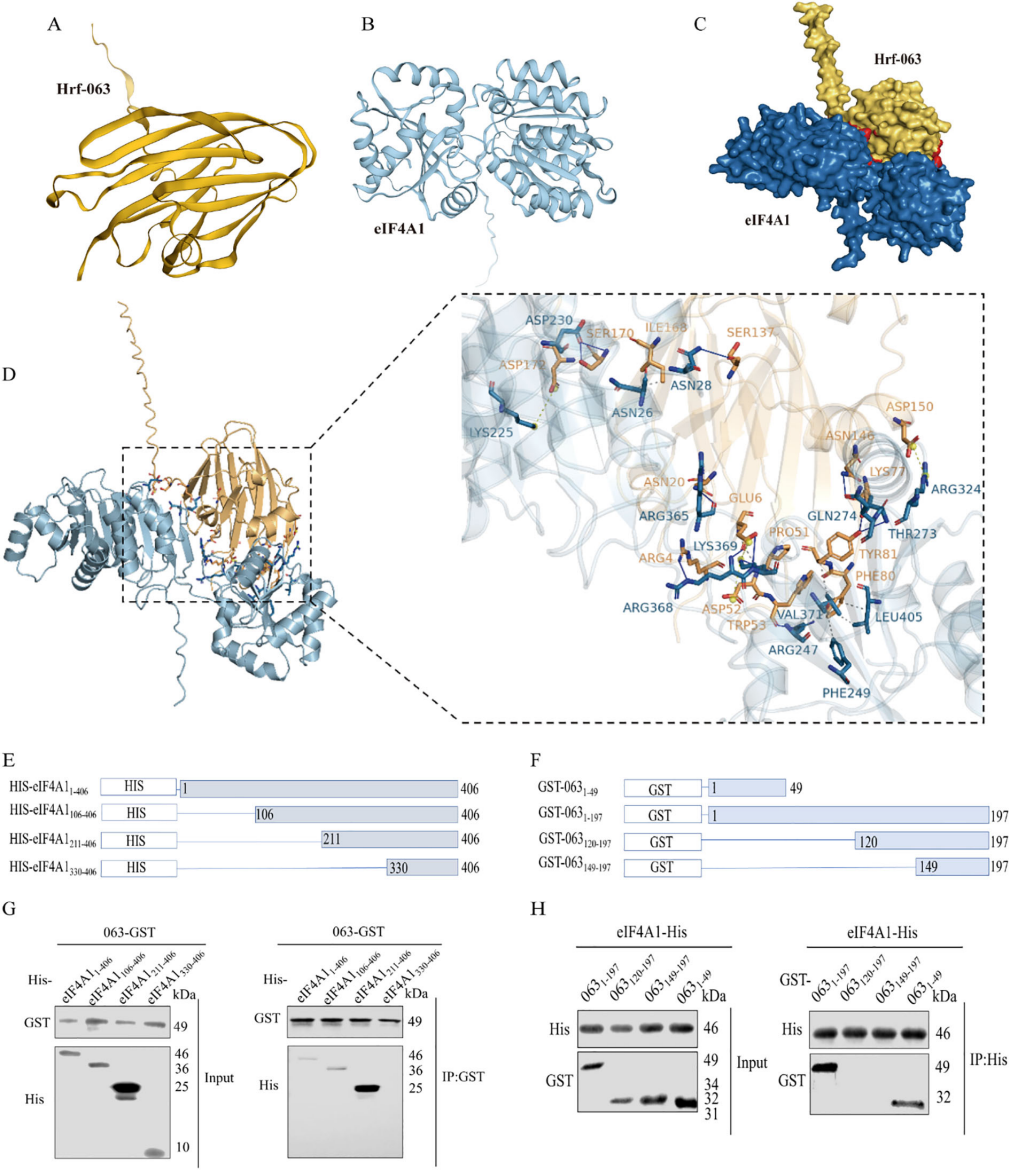
Prediction and mapping of the interaction regions between Hrf-063 and eIF4A1. **(A)** Predicted structure model of Hrf-063. **(B)** Predicted structure model of eIF4A1. **(C)** Surface representation of the modeled Hrf-063-eIF4A1 complex. Hrf-063 is shown in yellow and eIF4A1 in blue. The putative interaction interface is highlighted in red. **(D)** Ribbon representation of the modeled Hrf-063-eIF4A1 complex and enlarged view of the predicted binding interface. **(E)** Schematic representation of His-tagged eIF4A1 truncation constructs used for interaction mapping. **(F)** Schematic representation of GST-tagged Hrf-063 truncation constructs used for interaction mapping. **(G)** GST pull-down assay using GST-Hrf-063 as bait and the indicated His-tagged eIF4A1 truncations as prey. **(H)** His pull-down assay using His-eIF4A1 as bait and the indicated GST-tagged Hrf-063 truncations as prey.

To investigate the structural basis of the interaction between Hrf-063 and eIF4A1, an interaction model was generated using AlphaFold, and antigenic epitopes were further analyzed using DNASTAR. Based on the predicted epitope profiles, truncated protein constructs were generated for subsequent binding assays. Structural modeling indicated that the N-terminal region of Hrf-063 was predicted to interact with the 211–330 amino acid region of eIF4A1, forming a potential interaction interface (**Figures 3A–D**).

Subsequently, truncation constructs were generated according to the antigenic epitope profiles predicted by DNASTAR (**Figures 3E, F**). GST pull-down assays showed that the N-terminal fragment of Hrf-063 retained binding to eIF4A1, whereas the binding ability of the C-terminal truncation fragment was markedly weaker than that of the full-length protein. In the complementary localization assay, Hrf-063 exhibited strong binding to the 211–330 amino acid region of eIF4A1, whereas deletion of this region markedly weakened or abolished the interaction. Collectively, these data demonstrated that the N-terminal region of Hrf-063 and the 211–330 amino acid region of eIF4A1 were critical for mediating the interaction.

### 3.4 Hrf-063 and eIF4A1 co-localize predominantly in the cytoplasm

**Figure 4:**
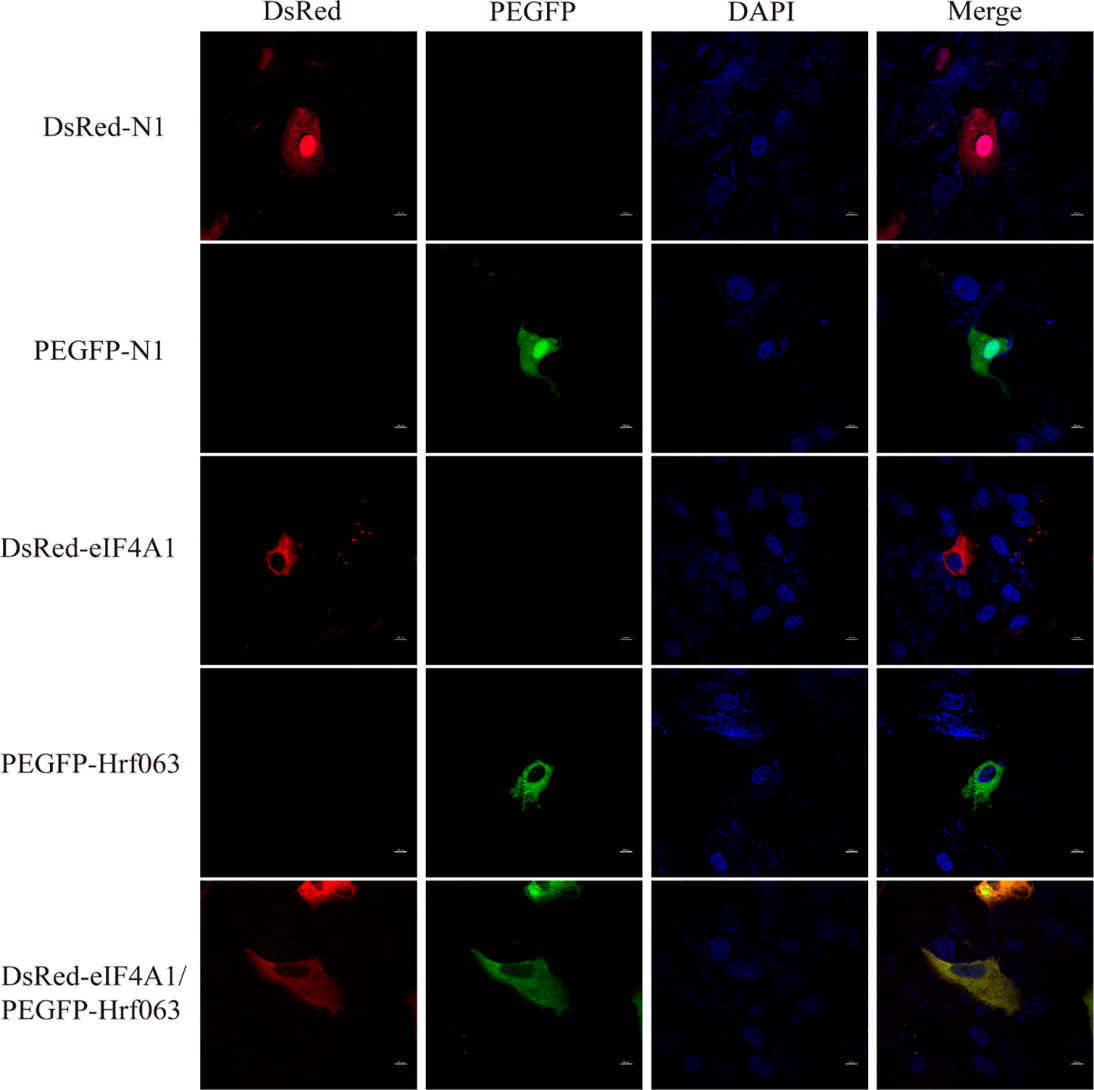
Subcellular co-localization of eIF4A1 and Hrf-063 in cells revealed by confocal laser scanning microscopy.

To determine whether Hrf-063 and eIF4A1 shared a common subcellular localization pattern, PEGFP-Hrf-063 and DsRed-eIF4A1 were co-expressed and examined by confocal microscopy. When the empty vectors DsRed-N1 and PEGFP-N1 were expressed alone, fluorescence was observed in both the nucleus and the cytoplasm, with stronger nuclear signals. In contrast, when expressed individually, both Hrf-063 and eIF4A1 were predominantly localized in the cytoplasm. Upon co-expression, substantial overlap of the two fluorescent signals was observed, giving rise to a clear merged signal in the same cellular regions (**Figure 4**). These findings were consistent with the biochemical interaction data and the cytoplasmic replication characteristics of GTPV.

### 3.5 Hrf-063 expression is associated with altered eIF4A1 levels, and eIF4A1 perturbation affects Klp2 expression during infection

**Figure 5:**
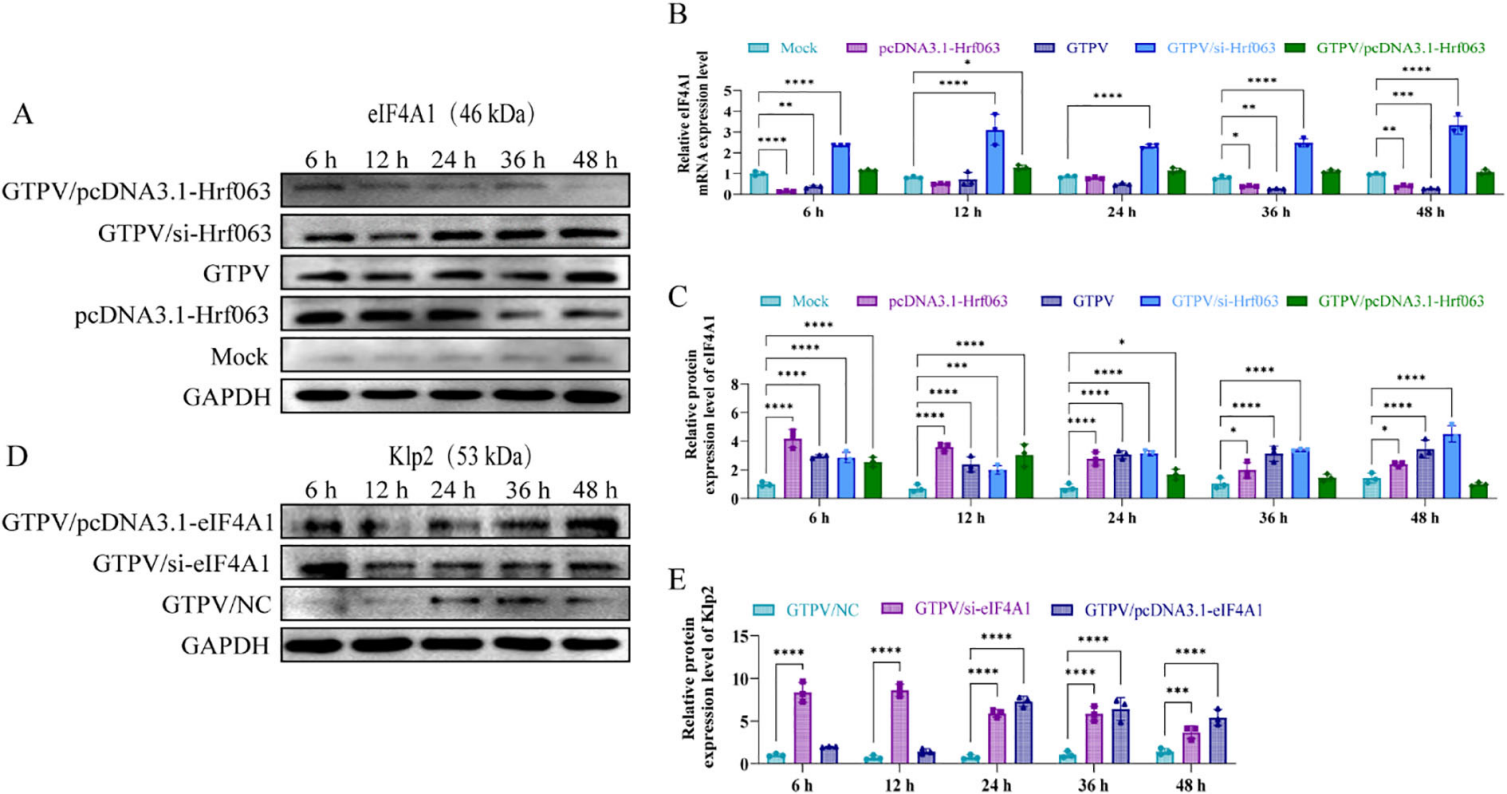
Effects of Hrf-063 and eIF4A1 on eIF4A1 mRNA/protein accumulation and Klp2 protein expression at different time points. **(A)** Western blot analysis of the eIF4A1 protein (46 kDa) was performed at 6, 12, 24, 36, and 48 hours under the specified treatment conditions (including GTPV/pcDNA3.1-Hrf063, GTPV/si-Hrf063, GTPV, pcDNA3.1-Hrf063, and Mock). GAPDH was used as an internal control. **(B)** Relative *eIF4A1* mRNA expression levels in samples treated with Mock, pcDNA3.1-Hrf063, GTPV, GTPV/si-Hrf063, and GTPV/pcDNA3.1-Hrf063 at 6, 12, 24, 36, and 48 hours. **(C)** Relative protein expression level of eIF4A1 corresponding to the treatments shown in **(A, D)** Immunoblot analysis of Klp2 protein (53 kDa) at 6, 12, 24, 36, and 48 h in GTPV/pcDNA3.1-eIF4A1, GTPV/si-eIF4A1, and GTPV/NC samples. GAPDH was used as the loading control. **(E)** Quantification of relative Klp2 protein expression level corresponding to panel D. Data in the bar charts are presented as mean ± SD. Asterisks indicate statistically significant differences as shown in the figure (*P < 0.05, **P < 0.01, ***P < 0.001, ****P < 0.0001).

The relationship between Hrf-063 and eIF4A1 expression was preliminarily examined at 6, 12, 24, 36, and 48 h post-infection. Under GTPV infection conditions, siRNA-mediated knockdown of *Hrf-063* increased *eIF4A1* mRNA expression levels across the analyzed time points, whereas Hrf-063 overexpression in infected cells reduced *eIF4A1* mRNA expression levels (**Figure 5B**). Western blot analysis revealed a similar overall trend at the protein level (**Figures 5A, C**): compared with the GTPV group, eIF4A1 protein abundance was generally increased in the GTPV/si-Hrf-063 group, but decreased in the GTPV/Hrf-063 overexpression group. In the absence of infection, Hrf-063 overexpression also markedly reduced eIF4A1 protein expression, suggesting that the regulatory trend of eIF4A1 in response to Hrf-063 was more pronounced than that observed under GTPV infection alone. Collectively, these findings supported a regulatory effect of Hrf-063 on eIF4A1 expression and a negative correlation between the two.

Kelch-like protein 2 (Klp2) is a virulence-associated factor of GTPV that influences viral pathogenicity in the host. To evaluate whether eIF4A1 was associated with the expression of viral proteins, eIF4A1 was subjected to knockdown or overexpression, and the expression level of GTPV Klp2 protein was subsequently examined. Compared with the infected control group, Klp2 expression was significantly increased upon eIF4A1 overexpression. In contrast, following eIF4A1 knockdown, Klp2 protein levels were significantly elevated relative to the control group at the early stage of infection, but showed a downward trend at later stages (**Figures 5D, E**). These findings indicated that eIF4A1 was associated with the expression of the GTPV virulence-related protein Klp2.

### 3.6 Hrf-063 and eIF4A1 were associated with differential regulation of STAT1 and CAV1 expression

**Figure 6:**
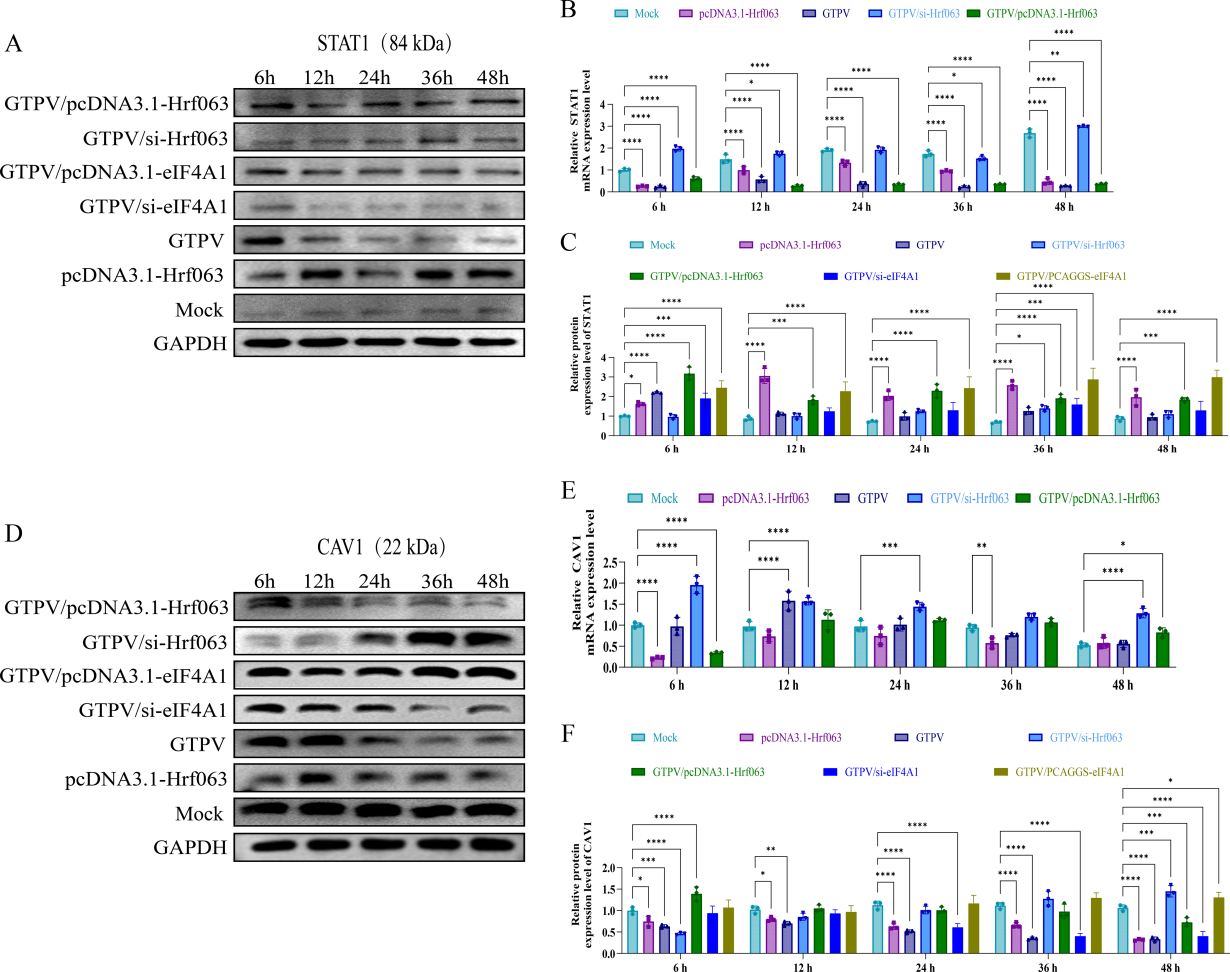
Effects of Hrf-063 and eIF4A1 on STAT1 and CAV1 expression at different time points. **(A)** Western blot analysis of the STAT1 protein (84 kDa) was performed at 6, 12, 24, 36, and 48 hours under the specified treatment conditions (including GTPV/pcDNA3.1-Hrf063, GTPV/si-Hrf063, GTPV, pcDNA3.1-Hrf063, and Mock). GAPDH was used as an internal control. **(B)** Relative *STAT1* mRNA expression levels at 6, 12, 24, 36, and 48 h in Mock, pcDNA3.1-Hrf063, GTPV, GTPV/si-Hrf063, and GTPV/pcDNA3.1-Hrf063 samples. **(C)** Relative protein expression level of STAT1 corresponding to the treatments shown in **(A, D)** Immunoblot analysis of CAV1 protein (22 kDa) at 6, 12, 24, 36, and 48 h in the same treatment groups shown in **(A)**. GAPDH was used as the loading control. **(E)** Relative *CAV1* mRNA expression levels at 6, 12, 24, 36, and 48 h in Mock, pcDNA3.1-Hrf063, GTPV, GTPV/si-Hrf063, and GTPV/pcDNA3.1-Hrf063 samples. **(F)** Quantification of relative CAV1 protein expression levels at 6, 12, 24, 36, and 48 h in Mock, pcDNA3.1-Hrf063, GTPV, GTPV/si-Hrf063, GTPV/pcDNA3.1-Hrf063, GTPV/si-eIF4A1, and GTPV/PCAGGS-eIF4A1 groups.Data in the bar charts are presented as mean ± SD. Asterisks indicate statistically significant differences as shown in the figure (*P < 0.05, **P < 0.01, ***P < 0.001, ****P < 0.0001).

STAT1 is a central component of antiviral signaling. To examine whether Hrf-063 and eIF4A1 were associated with changes in antiviral signaling-related factors, STAT1 expression was analyzed under different treatment conditions. In infected cells, silencing of *Hrf-063* increased *STAT1* mRNA expression, whereas Hrf-063 overexpression reduced *STAT1* mRNA expression (**Figure 6B**), indicating that Hrf-063 was negatively associated with STAT1 transcript abundance under infection conditions. Western blot analysis showed that Hrf-063 overexpression increased total STAT1 protein levels (**Figures 6A, C**). Under GTPV infection, total STAT1 protein levels increased significantly at the early stage and then gradually declined over time. Although Hrf-063 overexpression resulted in higher total STAT1 protein levels than those in the control group, total STAT1 protein expression still showed a decreasing trend over time under infection conditions. Silencing of *Hrf-063* also increased total STAT1 protein expression. In addition, silencing of *eIF4A1* reduced total STAT1 protein levels, whereas overexpression of eIF4A1 increased total STAT1 protein expression. Together, these findings suggest that STAT1 expression in this system may be influenced by additional post-transcriptional or translational mechanisms, and that Hrf-063 and eIF4A1 are associated with altered STAT1 abundance under the experimental conditions used in this study.

CAV1, a host factor associated with antiviral responses and membrane-related processes, was analyzed in parallel. Silencing of *Hrf-063* significantly increased *CAV1* mRNA levels, whereas Hrf-063 overexpression reduced *CAV1* transcription (**Figure 6E**). A similar overall trend was observed at the protein level, with Hrf-063 overexpression generally decreasing CAV1 protein abundance, whereas Hrf-063 silencing increased CAV1 protein expression. In contrast, modulation of eIF4A1 expression at the translational level produced the opposite pattern: *eIF4A1* silencing led to a downward trend in CAV1 protein expression, whereas eIF4A1 overexpression increased CAV1 protein levels (**Figures 6D, F**). These results indicate that both Hrf-063 and eIF4A1 contribute to the regulation of CAV1 expression.

### 3.7 Hrf-063 was associated with time-dependent changes in *ISG15* and *STING1* transcription during infection

**Figure 7:**
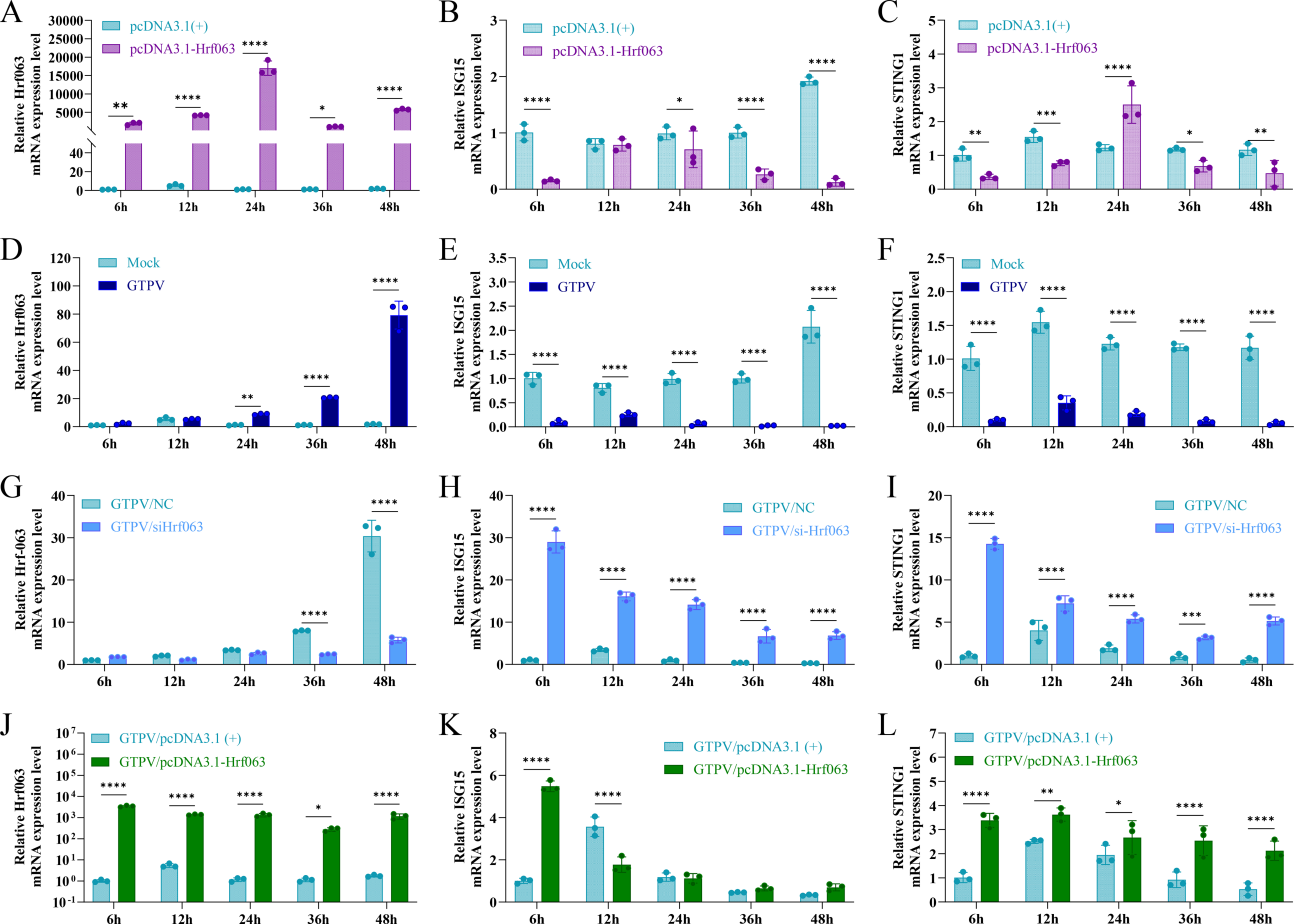
Effects of Hrf-063-related treatments on the mRNA expression levels of Hrf-063, *ISG15*, and *STING1* at different time points. **(A)** Relative *Hrf-063* mRNA expression levels at 6, 12, 24, 36, and 48 h in cells transfected with pcDNA3.1(+) or pcDNA3.1-Hrf063. **(B)** Relative *ISG15* mRNA expression levels at 6, 12, 24, 36, and 48 h in pcDNA3.1(+) and pcDNA3.1-Hrf063 groups. **(C)** Relative *STING1* mRNA expression levels at 6, 12, 24, 36, and 48 h in pcDNA3.1(+) and pcDNA3.1-Hrf063 groups. **(D)** Relative *Hrf-063* mRNA expression levels at 6, 12, 24, 36, and 48 h in Mock and GTPV groups. **(E)** Relative *ISG15* mRNA expression levels at 6, 12, 24, 36, and 48 h in Mock and GTPV groups. **(F)** Relative *STING1* mRNA expression levels at 6, 12, 24, 36, and 48 h in Mock and GTPV groups. **(G)** Relative *Hrf-063* mRNA expression levels at 6, 12, 24, 36, and 48 h in GTPV/NC and GTPV/si-Hrf063 groups. **(H)** Relative *ISG15* mRNA expression levels at 6, 12, 24, 36, and 48 h in GTPV/NC and GTPV/si-Hrf063 groups. **(I)** Relative *STING1* mRNA expression levels at 6, 12, 24, 36, and 48 h in GTPV/NC and GTPV/si-Hrf063 groups. **(J)** Relative *Hrf-063* mRNA expression levels at 6, 12, 24, 36, and 48 h in GTPV/pcDNA3.1(+) and GTPV/pcDNA3.1-Hrf063 groups. **(K)** Relative *ISG15* mRNA expression levels at 6, 12, 24, 36, and 48 h in GTPV/pcDNA3.1(+) and GTPV/pcDNA3.1-Hrf063 groups. **(L)** Relative *STING1* mRNA expression levels at 6, 12, 24, 36, and 48 h in GTPV/pcDNA3.1(+) and GTPV/pcDNA3.1-Hrf063 groups. Data are presented as mean ± SD. Statistical significance is indicated in the figure (*P < 0.05, **P < 0.01, ***P < 0.001, ****P < 0.0001).

To evaluate whether Hrf-063 affects the expression of innate immunity-related genes, the transcriptional levels of *ISG15* and *STING1* were analyzed under conditions of Hrf-063 overexpression and gene silencing. Expression of Hrf-063 alone significantly increased *Hrf-063* mRNA levels (**Figure 7A**), confirming the successful establishment of the expression system. Under these conditions, *ISG15* mRNA levels were reduced at most of the analyzed time points (**Figure 7B**). *STING1* exhibited a more complex pattern, with an overall downward trend but a transient increase within 24 h (**Figure 7C**). These observations suggested that expression of Hrf-063 alone was sufficient to alter the transcriptional profiles of selected innate immunity-related genes.

Under GTPV infection alone, *Hrf-063* transcript levels gradually increased over time (**Figure 7D**), whereas the mRNA levels of *ISG15* and *STING1* remained lower than those in control cells at all examined time points (**Figures 7E, F**). Importantly, compared with the infected control group, silencing of *Hrf-063* in infected cells restored the expression levels of both *ISG15* and *STING1* (**Figures 7H, I**), This supports the notion that Hrf-063 suppresses these genes during infection.

When *Hrf-063* was overexpressed under infection conditions, the expression patterns of *ISG15* and *STING1* again exhibited time-dependent changes. *ISG15* showed a downward trend (**Figure 7K**), whereas *STING1* expression was significantly increased relative to the control group at all examined time points (**Figure 7L**). These infection-dependent patterns suggested that the effects of Hrf-063 on innate immunity-related transcription were not strictly linear, but might depend on the stage of infection and the broader virus-host regulatory context. Therefore, the available data support an association between Hrf-063 expression and alterations in *ISG15* and *STING1* transcription, although the direct molecular mechanisms underlying these changes remain to be clarified.

### 3.8 Hrf-063 and eIF4A1 perturbation altered GTPV infectious titers

**Figure 8:**
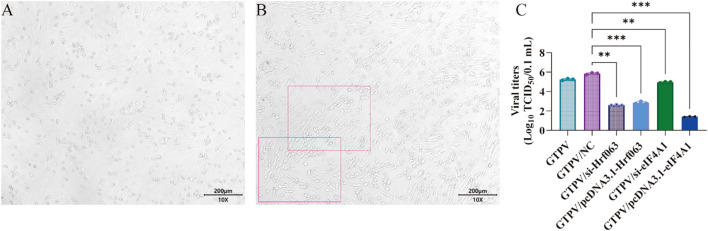
Effects of Hrf-063 and eIF4A1 perturbation on infectious GTPV titers. **(A)** Representative microscopic image of control cells without obvious cytopathic effects. Scale bars, 200 μm **(B)** Representative microscopic image of GTPV-infected cells showing cytopathic effects used for TCID_50_ determination. Scale bars, 200 μm. **(C)** Viral titers in GTPV-infected cells following Hrf-063 or eIF4A1 overexpression or knockdown. Virus-containing samples were harvested, subjected to three freeze–thaw cycles, serially diluted from 10^−1 to 10^−9, and titrated using the TCID_50_ assay. TCID_50_ values were calculated using the Kärber method and expressed as log_10_(TCID_50_/0.1 mL). Data are presented as the mean ± SD. Statistical significance was determined using one-way ANOVA followed by an appropriate *post hoc* test. **p < 0.01; ***p < 0.001.

To assess whether Hrf-063 and eIF4A1 affect infectious GTPV production, viral titers were determined using the TCID_50_ assay. GTPV-infected cells exhibited typical cytopathic effects compared with control cells (**Figures 8A, B**).

Virus-containing samples were serially diluted from 10^−1 to 10^−9, and viral titers were calculated using the Kärber method. The TCID_50_ values for the GTPV/pcDNA3.1-Hrf-063, GTPV/si-Hrf-063, GTPV/pcDNA3.1-eIF4A1, GTPV/si-eIF4A1, GTPV, and GTPV/NC groups were 10^−2.750, 10^−2.625, 10^−1.375, 10^−4.875, 10^−5.000, and 10^−5.750 per 0.1 mL, respectively (**Figure 8C**).

Compared with the GTPV and GTPV/NC groups, eIF4A1 overexpression reduced infectious viral titers, whereas eIF4A1 silencing partially restored viral titers (**Figure 8C**), suggesting that eIF4A1 was associated with reduced GTPV infectivity under the tested conditions. In addition, both overexpression and silencing of Hrf-063 resulted in decreased viral titers relative to the control groups, indicating that perturbation of Hrf-063 expression may impair efficient GTPV production (**Figure 8C**).

## 4 Discussion

GTPV infection is shaped by complex interactions between viral proteins and host cellular factors. Identifying host proteins targeted by viral gene products is therefore important for understanding viral replication, immune evasion, and pathogenicity (Zhao et al., 2019; Lu and Zhang, 2020; Shah et al., 2022; Boys et al., 2023; Sirihongthong and Auewarakul, 2025). In this study, we identified eIF4A1 as a candidate host factor that interacts with the GTPV Hrf-063 protein. This interaction was initially suggested by GST pull-down coupled with LC-MS/MS analysis and was subsequently validated by reciprocal pull-down and Co-IP assays. Domain-mapping and subcellular localization analyses further supported a biologically relevant association between Hrf-063 and eIF4A1. Functional assays showed that perturbation of Hrf-063 or eIF4A1 expression was associated with altered expression of viral and host immune-related factors, including Klp2, STAT1, CAV1, ISG15, and STING1, as well as changes in infectious viral titers. Collectively, these findings suggest that the Hrf-063-eIF4A1 axis may participate in the regulation of GTPV-host interactions.

In the initial screening, eIF4A1 was identified as the highest-ranking candidate among the Hrf-063-associated host proteins. The reliability of this candidate selection was supported by multiple LC-MS/MS quality-control parameters, including peptide length distribution, precursor mass error distribution, and cumulative protein identification profiles. Functional annotation of the identified proteins showed enrichment in translation-related processes, RNA binding, ATP binding, and ribosome- or cytoplasm-associated localization. These features are consistent with the known biological function of eIF4A1 as an ATP-dependent RNA helicase involved in translation initiation and RNA metabolism (Linder and Jankowsky, 2011; Taroncher-Oldenburg et al., 2021; Xue et al., 2021). Because poxviruses replicate in the cytoplasm and rely extensively on host translational machinery, the identification of a translation-associated host protein as an Hrf-063-interacting factor is biologically plausible (Grimm et al., 2022; Park and Walsh, 2022; Rozman et al., 2023). However, the pull-down and proteomic data alone cannot establish a direct functional role for eIF4A1 in GTPV infection. Therefore, biochemical and cellular validation experiments were necessary to confirm this interaction (Luck et al., 2020).

The interaction between Hrf-063 and eIF4A1 was supported by several complementary experimental approaches. Recombinant pull-down assays demonstrated that Hrf-063 and eIF4A1 could associate under *in vitro* conditions, whereas Co-IP assays confirmed that this interaction also occurred in mammalian cells. The reciprocal pull-down and Co-IP experiments strengthened the evidence for a specific association between these two proteins. In addition, confocal microscopy showed that Hrf-063 and eIF4A1 were predominantly localized in the cytoplasm and displayed substantial signal overlap upon co-expression. This localization pattern is consistent with the cytoplasmic replication cycle of GTPV and provides spatial support for the observed biochemical interaction (Tolonen et al., 2001; Katsafanas and Moss, 2007; Moss, 2013). Nevertheless, fluorescence co-localization does not by itself demonstrate direct physical binding (Dunn et al., 2011; Aaron et al., 2018). Rather, together with the pull-down and Co-IP results, it supports the conclusion that Hrf-063 and eIF4A1 are present in the same cellular compartment and are capable of forming a protein complex (Varnaitė and MacNeill, 2016).

Domain-mapping analysis further indicated that the N-terminal region of Hrf-063 and the 211–330 amino acid region of eIF4A1 were important for their interaction. AlphaFold-based structural prediction suggested a potential interaction interface between these regions, and truncation-based binding assays provided experimental support for this model. The reduced binding capacity of the Hrf-063 C-terminal fragment, together with the strong binding observed for the Hrf-063 N-terminal region, suggests that the N terminus of Hrf-063 contains key determinants required for eIF4A1 association. Similarly, deletion or truncation affecting the 211–330 amino acid region of eIF4A1 markedly weakened the interaction, indicating that this region contributes substantially to Hrf-063 binding. These findings provide a structural basis for future mechanistic studies. For example, site-directed mutagenesis within the predicted interface could help identify specific residues required for this interaction and clarify whether disruption of the Hrf-063-eIF4A1 interaction affects viral replication or immune modulation.

An important observation in this study was that Hrf-063 expression was negatively associated with eIF4A1 expression. During GTPV infection, Hrf-063 knockdown increased eIF4A1 mRNA and protein levels, whereas Hrf-063 overexpression reduced eIF4A1 expression. This trend was also evident in the absence of infection, in which Hrf-063 overexpression markedly reduced eIF4A1 protein abundance. These findings suggest that Hrf-063 may regulate eIF4A1 expression either directly or indirectly. Given that eIF4A1 is involved in translation initiation, downregulation of eIF4A1 by a viral protein may represent a strategy to reshape host protein synthesis during infection (Tauber et al., 2020; Burgess et al., 2022; Imai et al., 2023; Du et al., 2025). However, the current data do not distinguish whether Hrf-063 affects eIF4A1 transcription, mRNA stability, protein synthesis, or protein degradation. Therefore, additional experiments, such as promoter activity assays, mRNA decay analysis, cycloheximide chase assays, and proteasome or lysosome inhibition assays, would be required to define the precise regulatory mechanism (Foster et al., 2006; Klionsky et al., 2021; Li et al., 2021).

The relationship between eIF4A1 and viral protein expression appeared to be complex. eIF4A1 overexpression increased the expression of the GTPV virulence-associated protein Klp2, suggesting that eIF4A1 may facilitate the expression of at least some viral proteins. In contrast, eIF4A1 knockdown increased Klp2 protein levels at the early stage of infection but was associated with a decreasing trend at later stages. This time-dependent pattern indicates that eIF4A1 may exert stage-specific effects during infection (Kakuk et al., 2023). One possible interpretation is that early viral gene expression and later viral protein accumulation may differ in their dependence on host translation factors (Soday et al., 2019; Deng et al., 2025; Yi et al., 2026). Alternatively, compensatory cellular responses triggered by eIF4A1 depletion may transiently affect viral protein synthesis (DiGiuseppe et al., 2020). Because Klp2 was used here as a representative virulence-associated viral protein, these results should not be generalized to all GTPV proteins without further analysis. Global viral transcriptomic and proteomic profiling would be useful to determine whether eIF4A1 broadly regulates viral gene expression or selectively influences specific viral proteins (Bojkova et al., 2020; Bian et al., 2025).

Perturbation of Hrf-063 and eIF4A1 also affected the expression of STAT1, a key component of antiviral signaling. At the transcript level, Hrf-063 knockdown increased STAT1 mRNA expression, whereas Hrf-063 overexpression reduced STAT1 mRNA expression during infection. These findings suggest that Hrf-063 is negatively associated with STAT1 transcript abundance under the tested conditions. At the protein level, however, the regulatory pattern was more complex. Hrf-063 overexpression increased total STAT1 protein levels, and Hrf-063 knockdown also elevated STAT1 protein abundance. In addition, eIF4A1 knockdown reduced total STAT1 protein levels, whereas eIF4A1 overexpression increased STAT1 protein expression. Thus, changes in STAT1 protein abundance were not always concordant with changes in STAT1 mRNA levels (Liu et al., 2016). This discrepancy suggests that STAT1 expression may be regulated at multiple levels, including transcriptional, post-transcriptional, translational, and post-translational mechanisms (Buccitelli and Selbach, 2020). It is also important to note that the present study measured total STAT1 protein rather than phosphorylated STAT1 or STAT1 nuclear translocation. Therefore, increased total STAT1 abundance does not necessarily indicate enhanced STAT1 signaling activity (Hu and Ivashkiv, 2009). Future studies should examine phosphorylated STAT1, interferon-stimulated gene activation, and STAT1-dependent antiviral responses to determine how Hrf-063 and eIF4A1 influence the functional status of the STAT1 pathway.

CAV1 was also differentially regulated by Hrf-063 and eIF4A1. Hrf-063 knockdown increased CAV1 mRNA and protein expression, whereas Hrf-063 overexpression generally reduced CAV1 abundance. These results suggest that Hrf-063 may suppress CAV1 expression during infection. In contrast, eIF4A1 overexpression increased CAV1 protein levels, whereas eIF4A1 knockdown reduced CAV1 protein abundance. This opposite pattern indicates that Hrf-063 and eIF4A1 may exert distinct or even antagonistic effects on CAV1 regulation. CAV1 is involved in membrane organization, endocytosis, and cellular signaling, and these processes may influence viral entry, assembly, egress, or host antiviral responses (Cai et al., 2008; Parton and del Pozo, 2013; Zhang et al., 2024). The observed regulation of CAV1 therefore provides a potential link between the Hrf-063-eIF4A1 interaction and membrane-associated host responses. However, whether CAV1 directly affects GTPV replication or acts as part of a broader antiviral response remains to be determined.

The effects of Hrf-063 on ISG15 and STING1 transcription further suggest that this viral protein may participate in the modulation of innate immune responses. Hrf-063 expression alone reduced ISG15 mRNA levels at most time points and produced a more dynamic pattern of STING1 transcription. During GTPV infection, ISG15 and STING1 transcript levels were lower than those in control cells, whereas Hrf-063 silencing restored their expression. These data support the possibility that Hrf-063 contributes to the suppression of selected innate immunity-related genes during infection. However, when Hrf-063 was overexpressed during infection, ISG15 showed a decreasing trend, whereas STING1 expression increased at all examined time points. This result indicates that the regulatory effect of Hrf-063 on innate immune gene transcription is not strictly linear and may depend on the stage of infection, viral replication status, or broader cellular immune context. In particular, STING1 expression may be influenced by feedback regulation within DNA-sensing pathways, cellular stress responses, or secondary effects of altered viral replication (Pan et al., 2023; Zhang et al., 2024; Zhang and Zhang, 2025). Therefore, although the current data support an association between Hrf-063 expression and altered ISG15/STING1 transcription, further studies are needed to determine whether Hrf-063 directly targets these pathways.

The viral titer data provided functional evidence that both Hrf-063 and eIF4A1 are associated with infectious GTPV production. eIF4A1 overexpression reduced infectious viral titers, whereas eIF4A1 knockdown partially restored viral titers compared with the infected control groups. This result suggests that eIF4A1 may restrict infectious GTPV production under the experimental conditions used in this study, despite its positive association with Klp2 expression. This apparent discrepancy may reflect differences between the expression of an individual viral protein and the production of infectious progeny virus (de Wilde et al., 2018). Efficient viral propagation requires coordinated viral genome replication, gene expression, virion assembly, maturation, and release (V’Kovski et al., 2021). Therefore, a host factor that enhances the expression of one viral protein may still negatively affect other steps of the viral life cycle (Stern-Ginossar et al., 2019). In addition, eIF4A1 may influence host antiviral responses, cellular viability, or translational balance, all of which could affect infectious virus yield.

Interestingly, both overexpression and knockdown of Hrf-063 reduced viral titers relative to the control groups. This finding suggests that appropriate regulation of Hrf-063 expression may be important for efficient GTPV production. Excessive Hrf-063 expression may disrupt the balance of host-virus interactions or trigger unfavorable cellular responses, whereas Hrf-063 depletion may impair viral functions required for productive infection. Such a pattern is consistent with the idea that viral proteins often function within a tightly regulated temporal and quantitative context (Guo et al., 2020). However, because overexpression and siRNA-mediated knockdown can introduce non-physiological effects, these results should be interpreted with caution. Rescue experiments using siRNA-resistant Hrf-063, recombinant viruses carrying targeted mutations in Hrf-063, and analyses of viral DNA replication and progeny formation would help clarify the precise role of Hrf-063 in the GTPV life cycle.

Overall, this study identifies eIF4A1 as a host protein associated with GTPV Hrf-063 and provides evidence that this interaction is linked to changes in viral protein expression, host antiviral signaling-related factors, innate immunity-related gene transcription, and infectious viral production. The data support a working model in which Hrf-063 interacts with eIF4A1 in the cytoplasm and modulates host translation- and immunity-associated pathways during infection. Through this interaction, Hrf-063 may contribute to the regulation of eIF4A1 expression and influence downstream factors such as Klp2, STAT1, CAV1, ISG15, and STING1. Nevertheless, the current study primarily establishes association and functional correlation. The direct molecular mechanisms by which Hrf-063 regulates eIF4A1 and immune-related pathways remain to be elucidated.

Several limitations should be considered. First, although multiple biochemical assays validated the Hrf-063-eIF4A1 interaction, the exact amino acid residues mediating this interaction were not identified. Second, this study analyzed total STAT1 rather than activated STAT1 signaling, limiting conclusions regarding antiviral pathway activity. Third, the regulation of ISG15 and STING1 was examined at the transcriptional level, whereas corresponding protein levels and pathway activation markers were not assessed. Fourth, viral titer analysis demonstrated changes in infectious virus production but did not define which stage of the viral life cycle was affected. Finally, overexpression and knockdown approaches may alter cellular homeostasis; therefore, future studies using recombinant viruses and rescue systems will be important for confirming the physiological relevance of these findings.

In conclusion, our findings reveal a previously uncharacterized interaction between GTPV Hrf-063 and host eIF4A1. The Hrf-063-eIF4A1 interaction may represent an important component of GTPV-host interplay, linking viral protein function with host translational regulation and innate immune responses. These results provide new insight into the molecular mechanisms underlying GTPV infection and identify eIF4A1 as a potential host factor involved in the regulation of viral replication and antiviral signaling. Further mechanistic studies will be required to determine whether disruption of the Hrf-063-eIF4A1 interaction can be exploited as a strategy for controlling GTPV infection.

## Data Availability

The datasets presented in this study can be found in online repositories. The names of the repository/repositories and accession number(s) can be found in the article/Supplementary Material.
